# Resistance to Rabies

**DOI:** 10.4269/ajtmh.2012.12-0304

**Published:** 2012-08-01

**Authors:** Rodney E. Willoughby

**Affiliations:** Department of Pediatrics, Medical College of Wisconsin, Milwaukee, Wisconsin

Rabies is rapidly progressive, an almost universally fatal encephalitis caused by lyssaviruses, most notably rabies virus. Canine rabies is estimated to kill about 55,000 persons annually in Africa and Asia alone^1^; for different reasons, rabies is newly emerging in China, the former Soviet Republics, southern Africa, and Central and South America.

The article in this issue by Gilbert and colleagues^2^ challenges the orthodoxy that rabies is untreatable and universally fatal. This rule of perfect fatality has never been true for many mammalian species, whereas others, like the fox and man, succumb quickly. This same group recently provided compelling evidence that some bat species, known reservoirs of rabies, regularly recover from rabies.^3^

If complete or relative resistance to rabies were to occur in humans, it should be detected sporadically, such as in the recent well-characterized reports of rabies survivors in Texas and California.^4^,^5^ Although the illnesses in these patients were highly dissimilar in spectrum and severity, both children developed antibodies to cytoplasmic components of the rabies virus without neutralizing antibodies to the membrane-associated rabies glycoprotein. These case reports challenge one of two orthodoxies in the rabies field: either rabies is not always neurologically severe, or rabies can be cleared non-cytolytically (i.e., with minimal neurological damage) by mechanisms other than neutralizing antibodies to the rabies virus glycoprotein. Other hypotheses to explain these cases, such as autoimmune encephalitides cross-reactive with rabies virus structural proteins, were not supported by clinical tests.^6^

If complete or relative resistance to rabies were to exist in distinct populations, that would then likely require prolonged selection in a rabies-endemic region of genetically isolated human populations. Where would one look? One might postulate remote areas with small indigenous populations such as the Arctic (where fox rabies virus is endemic) or the Amazon, Africa, or Asia where bat or carnivore rabies viruses, and probably other bat-associated lyssaviruses, are endemic. The article in this issue by Gilbert brings us much closer to this possibility. It is notable that subjects in the Peruvian Amazon had antibodies to either the rabies virus nucleoprotein (as did survivors in the United States) or neutralizing antibodies to the membrane glycoprotein, but rarely to both. It is also notable that all seropositive subjects were adults, suggesting a fitness advantage to rabies resistance in a region where child survival is poor and 80% of subjects suffered depredation by vampire bats.

Where does this leave the rest of humankind who are highly susceptible to rabies? Rabies has been vaccine-preventable for over a century; the economic and geographic maldistributions of rabies vaccines and immunoglobulins are indefensible. Technology transfer will hopefully improve this with time. Gilbert has identified an opportunity for novel therapeutics, discoverable through whole genome sequencing. Careful, respectful genetic study of these genetically unique populations may provide information on which pathways in human biochemistry and physiology promote resistance to human rabies. Equally important, knowing that there is a continuum of disease, even for infectious diseases like rabies, should push us harder to try for cures when confronted by so-called untreatable infectious diseases or intoxications. Modern therapeutics can move us along a continuum toward greater survival, even when specific cures or antidotes remain undiscovered.
